# A Novel Conserved Linear Neutralizing Epitope on the Receptor-Binding Domain of the SARS-CoV-2 Spike Protein

**DOI:** 10.1128/spectrum.01190-23

**Published:** 2023-06-12

**Authors:** Rong-Hong Hua, Shu-Jian Zhang, Bei Niu, Jin-Ying Ge, Ting Lan, Zhi-Gao Bu

**Affiliations:** a State Key Laboratory for Animal Disease Control and Prevention, Harbin Veterinary Research Institute of Chinese Academy of Agricultural Sciences, Harbin, China; Chinese Academy of Sciences Wuhan Institute of Virology

**Keywords:** SARS-CoV-2, spike protein, receptor-binding domain, neutralizing antibody, epitope

## Abstract

The continuous emergence of new variants of severe acute respiratory syndrome coronavirus 2 (SARS-CoV-2) has made it challenging to develop broad-spectrum prophylactic vaccines and therapeutic antibodies. Here, we have identified a broad-spectrum neutralizing antibody and its highly conserved epitope in the receptor-binding domain (RBD) of the spike protein (S) S1 subunit of SARS-CoV-2. First, nine monoclonal antibodies (MAbs) against the RBD or S1 were generated; of these, one RBD-specific MAb, 22.9-1, was selected for its broad RBD-binding abilities and neutralizing activities against SARS-CoV-2 variants. An epitope of 22.9-1 was fine-mapped with overlapping and truncated peptide fusion proteins. The core sequence of the epitope, ^405^D(N)EVR(S)QIAPGQ^414^, was identified on the internal surface of the up-state RBD. The epitope was conserved in nearly all variants of concern of SARS-CoV-2. MAb 22.9-1 and its novel epitope could be beneficial for research on broad-spectrum prophylactic vaccines and therapeutic antibody drugs.

**IMPORTANCE** The continuous emergence of new variants of SARS-CoV-2 has caused great challenge in vaccine design and therapeutic antibody development. In this study, we selected a broad-spectrum neutralizing mouse monoclonal antibody which recognized a conserved linear B-cell epitope located on the internal surface of RBD. This MAb could neutralize all variants until now. The epitope was conserved in all variants. This work provides new insights in developing broad-spectrum prophylactic vaccines and therapeutic antibodies.

## INTRODUCTION

Since the first outbreak of coronavirus disease 2019 (COVID-19) at the end of 2019 (1), severe acute respiratory syndrome coronavirus 2 (SARS-CoV-2) has resulted in more than 700 million confirmed infections and 6.8 million deaths as of February 2023, according to data released by the WHO (https://covid19.who.int/). Numerous neutralizing antibodies targeting SARS-CoV-2 have been developed and have shown promise in clinical studies (2–4). However, due to the constantly mutating nature of the virus that confers emerging variants with immune escape abilities (5–7), the development of broad-spectrum therapeutic antibody drugs and prophylactic vaccines is still challenging.

SARS-CoV-2 belongs to the *Betacoronavirus* genus of the *Coronaviridae* family. The virion has four structural proteins (spike, S; envelope, E; matrix, M; and nucleocapsid, N), among which the S protein plays important roles in receptor binding-mediated cell invasion and induction of protective neutralizing antibodies (8–10). The S protein, a typical class I viral fusion protein, is present on the surface of virions in the trimeric form and consists of two functionally distinct subunits (S1 and S2). The S1 subunit contains an N-terminal domain and a receptor-binding domain (RBD). The S2 subunit contains a fusion peptide and two-heptad repeat regions, which mediate viral cell membrane fusion by forming a six-helix bundle (11–13). Neutralizing antibodies targeting different subdomains on the S protein can prevent SARS-CoV-2 infection, and accordingly, neutralizing antibodies targeting the RBD, N-terminal domain (NTD), and S2 have been reported for potential therapeutic use (4). The RBD is responsible for receptor angiotensin converting enzyme 2 (ACE2) binding, which mediates subsequent viral uptake and fusion (10). Accordingly, potent neutralizing antibodies against SARS-CoV-2 mostly target the RBD (14). However, since the first emergence of SARS-CoV-2, continuous mutations have occurred in the S protein, especially in the RBD. Distinct variants of concern (VOCs) or variants of interest carry mutations associated with enhancement of human-to-human transmission or neutralizing antibody escape (5, 7, 15–17). Therefore, to overcome the improved immune escape abilities of these mutations, it is necessary to generate and screen broad-spectrum neutralizing antibodies and identify highly conserved epitopes for the design and development of therapeutic drugs and preventive vaccines.

In the present study, through *in vitro* experiments on mammalian cell lines, we generated a monoclonal antibody (MAb) against RBD that had broad binding abilities and neutralizing activities against several SARS-CoV-2 variants, and we also fine-mapped a novel, highly conserved epitope of the MAb with overlapping peptide and truncated peptide fusion proteins and elucidated its core sequence.

## RESULTS

### Generation and characterization of MAbs.

**FIG 1:**
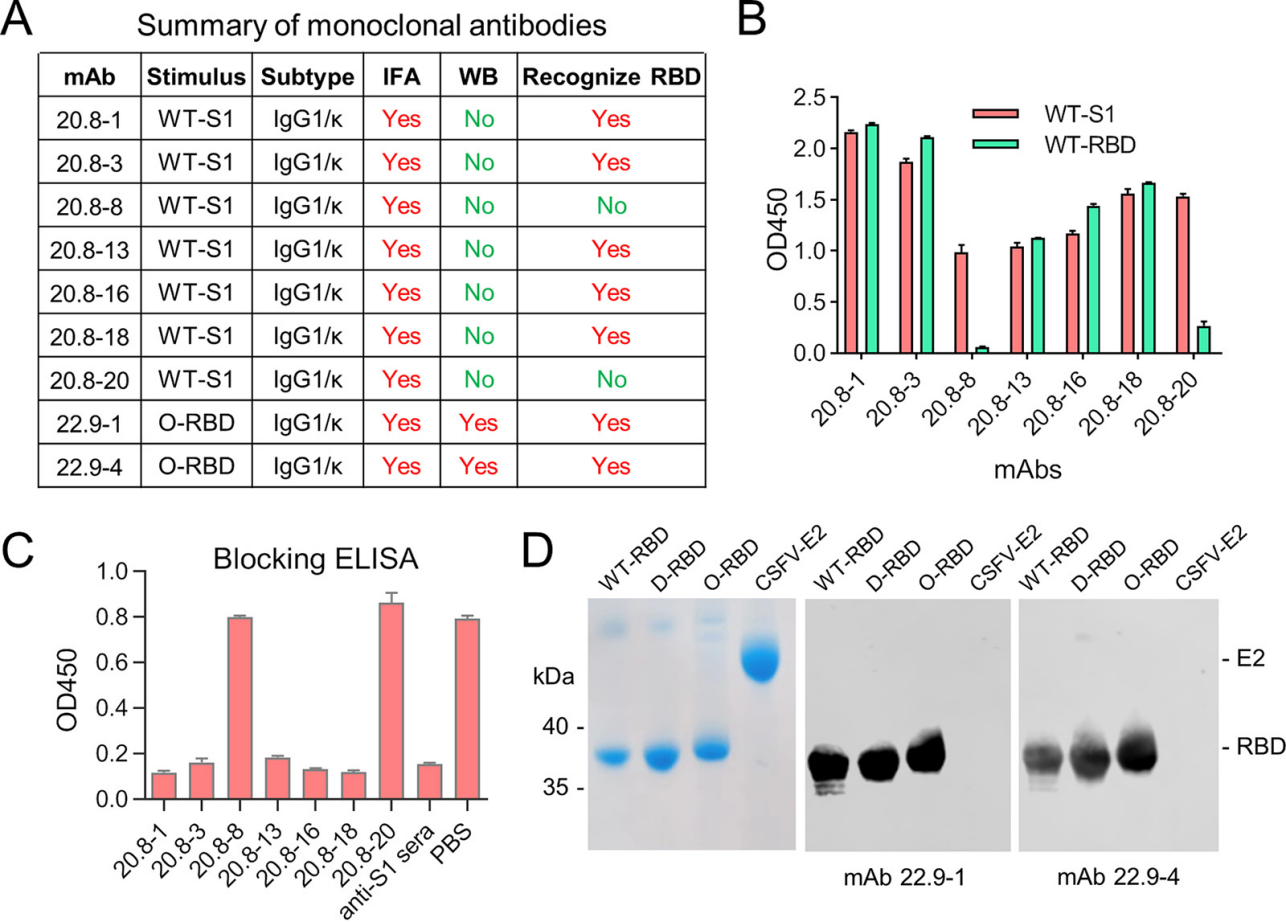
Generation and characterization of monoclonal antibodies against SARS-CoV-2. (A) A total of nine monoclonal antibodies were generated. Seven were derived from WT-S1, and two were derived from O-RBD. Heavy chain and light chain subtypes and the reactivities of the monoclonal antibodies were determined by immunofluorescence and Western blotting assays. (B) The reactivities of seven WT-S1-derived monoclonal antibodies against WT-S1 and WT-RBD were examined by ELISA. (C) Using HRP-conjugated MAb 20.8-1 as the detecting antibody, blockage of the binding of WT-S1 to MAb 20.8-1 was examined with the indicated MAbs. All five MAbs that recognized WT-RBD also recognized the same epitope. (D) Western blot detection of RBD variants that reacted with MAb 22.9-1 and MAb 2.9–4.

We first expressed and purified wild-type (WT; SARS-CoV-2 strain Wuhan-Hu-1), S1 protein, and Delta RBD proteins (see Fig. S1 in the supplemental material). The experimental mice were then immunized with WT-S1, and after cell fusion, seven strains of hybridoma cell lines were generated and selected. The seven monoclonal antibodies extracted from these cell lines were all characterized by the IgG1 isotype, and the light chains of the seven monoclonal antibodies were all kappa (Fig. 1A). An immunofluorescence assay (IFA) showed that all seven MAbs recognized native S protein in cells, but Western blot analysis showed that none of the seven MAbs reacted with denatured S1 protein in the lysate of cells. To explore whether the generated MAbs recognized the RBD, we examined their reactivity with WT-RBD by enzyme-liked immunosorbent assay (ELISA). The results showed that five of the seven MAbs recognized both WT-RBD and WT-S1, while two MAbs (20.8-8 and 20.8-20) recognized WT-S1 only (Fig. 1B). A blocking ELISA of the five MAbs that recognized WT-RBD indicated that all five MAbs recognized the same epitope as MAb 20.8-1 (Fig. 1C). 20.8-1 was selected out of the five for further investigation.

The RBD of the Omicron variant (O-RBD) was expressed, purified, and used as an immunogen to generate two more MAbs, namely, 22.9-1 and 22.9-4 (Fig. 1A). Western blotting results showed that both 22.9-1 and 22.9-4 recognized native and denatured RBD protein, and they also broadly reacted with WT-RBD, D-RBD, and O-RBD (Fig. 1D).

### RBD-binding ability of the generated MAbs.

**FIG 2:**
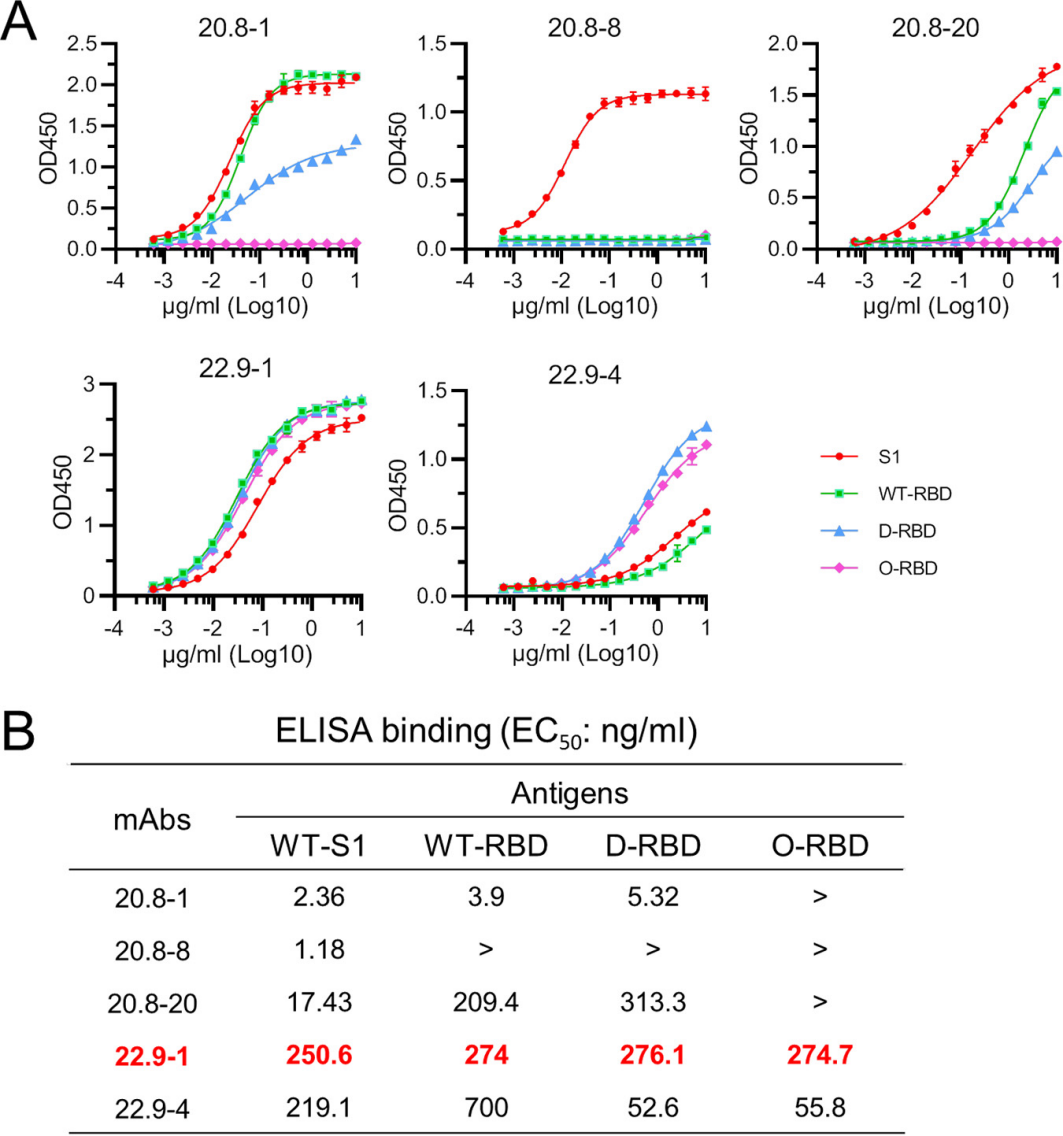
Binding of MAbs with WT-S1 and RBD variants. (A) The binding of five MAbs with the WT-S1 protein and three RBD variants was characterized by ELISA. Only MAb 22.9-1 reacted with all four tested proteins. (B) The EC_50_ values for ELISA were calculated with GraphPad Prism. The EC_50_ values of MAb 22.9-1 for binding with S1 and the three RBD variants ranged from 250.6 ng/mL to 276.1 ng/mL. Data from one of two independent experiments performed in triplicate are shown.

The RBD-binding ability of MAbs was examined using ELISA. While all the MAbs reacted with WT-S1, three of the MAbs did not react with O-RBD. Further, MAb 20.8-8 and MAb 20.8-20 showed different binding profiles. That is, 20.8-8 reacted with only WT-S1, and 20.8-20 only weakly reacted with WT-RBD and D-RBD at relatively high concentrations (Fig. 2A). Both O-RBD-derived MAbs, that is, 22.9-1 and 22.9-4, reacted with all the tested antigens (Fig. 2A). Although MAb 22.9-4 showed broad reactivity and had relatively low concentrations for the 50% maximal effect concentration (OD_450_) for its reaction with WT-RBD and WT-S1, the optical density at 450 nm (OD_450_) values were fairly low, even at a high antibody concentration of 10 μg/mL. In contrast, MAb 22.9-1 showed comparable binding profiles for WT-S1, WT-RBD, D-RBD, and O-RBD, with EC_50_ values ranging from 250.6 ng/mL to 276.1 ng/mL (Fig. 2B). These results indicated that MAb 22.9-1 cross-reacted with WT-RBD, D-RBD, and O-RBD. To further characterize the binding of MAb 22.9-1 to RBDs, the binding affinities of MAb 22.9-1 for WT-RBD, D-RBD, and O-RBD were determined with surface plasmon resonance (SPR). MAb 22.9-1 bound to O-RBD, WT-RBD, and D-RBD with *K_D_* (equilibrium dissociation constant) values of 117 nM, 339 nM, and 442 nM, respectively (Fig. S2).

### Neutralization activities of MAbs against pseudovirus.

**FIG 3:**
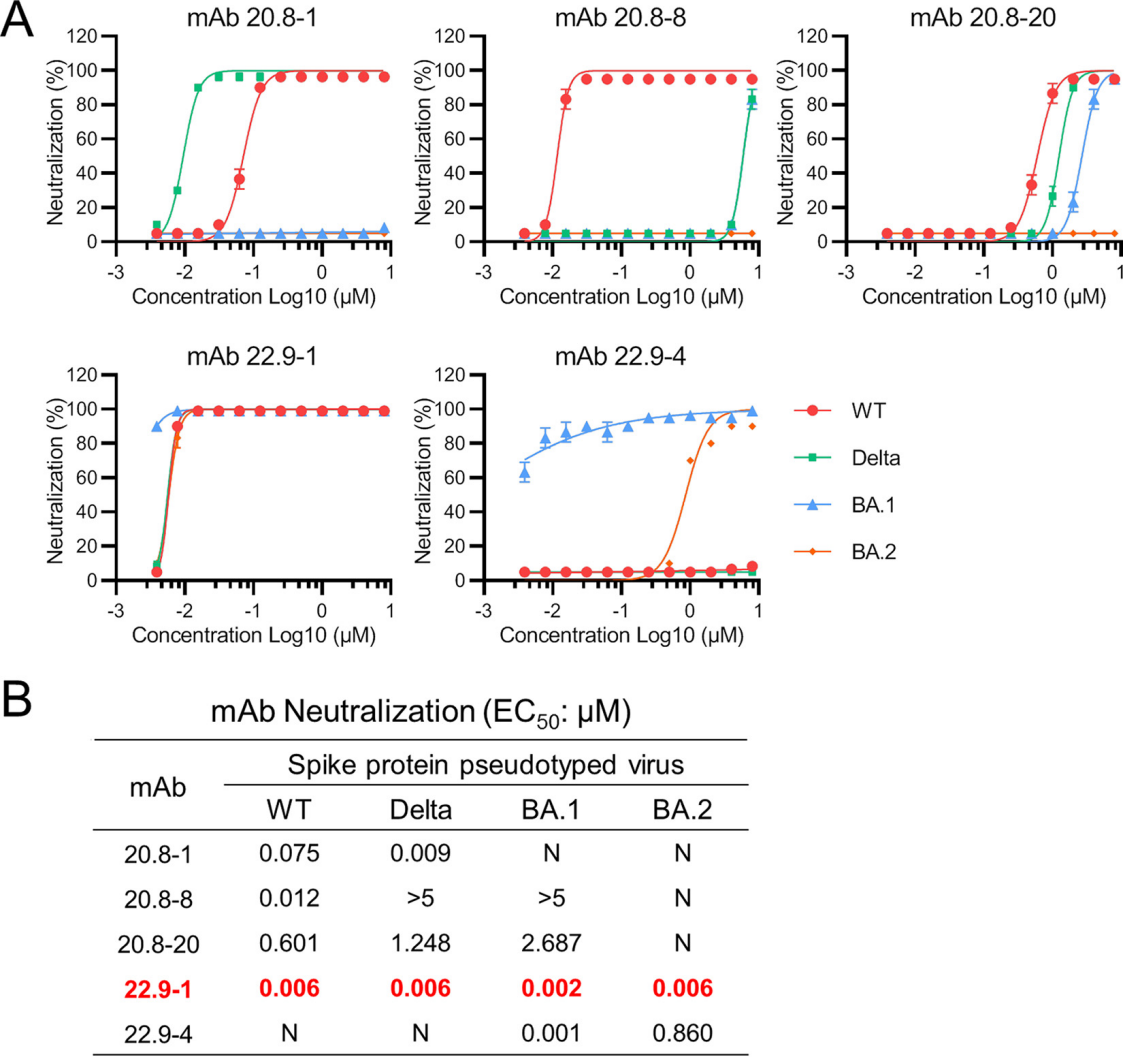
Neutralizing activity of monoclonal antibodies against SARS-CoV-2 pseudovirus. SARS-CoV-2 pseudovirus was incubated with 2-fold serially diluted monoclonal antibodies. The mixtures were then added to Vero E6 cells and incubated for 24 h, after which the neutralization potencies of the MAbs were evaluated (A). EC_50_ values were calculated with GraphPad Prism (B). Among the five tested MAbs, MAb 22.9-1 showed broad neutralizing activity against all four variants. Data from one of two independent experiments performed in triplicate are shown.

The neutralization activities of five MAbs were measured against four types of chimeric virus created by pseudotyping VSVΔG-GFP-nCoVS with WT, Delta, Omicron BA.1 (BA.1), and Omicron BA.2 (BA.2) spike protein. None of the three MAbs derived from WT-S1 neutralized the BA.2 pseudovirus (Fig. 3A). MAb 20.8-1 neutralized the WT and Delta pseudovirus at EC_50_s of 0.075 μM and 0.009 μM, respectively (Fig. 3B). MAb 20.8-8 neutralized only the WT pseudovirus, and MAb 20.8-20 showed weak neutralizing activity against the WT, Delta, and BA.1 pseudovirus (Fig. 3A). Both MAbs (22.9-1 and 22.9-4) derived from O-RBD could neutralize the BA.1 and BA.2 pseudovirus (Fig. 3A). However, MAb 22.9-4 showed no neutralization activity against the WT and Delta pseudovirus. Interestingly, MAb 22.9-1 exhibited broad neutralization activity against all four pseudoviruses (Fig. 3A), with EC_50_ values of 2 nM against the BA.1 pseudovirus and 6 nM against the WT, Delta, and BA.2 pseudovirus (Fig. 3B). Thus, a monoclonal antibody with broad neutralizing activity against SARS-CoV-2 variants was generated, and the results also indicated that there was a conserved linear epitope in the RBD of SARS-CoV-2 variants.

### Epitope mapping of MAb 22.9-1.

**FIG 4:**
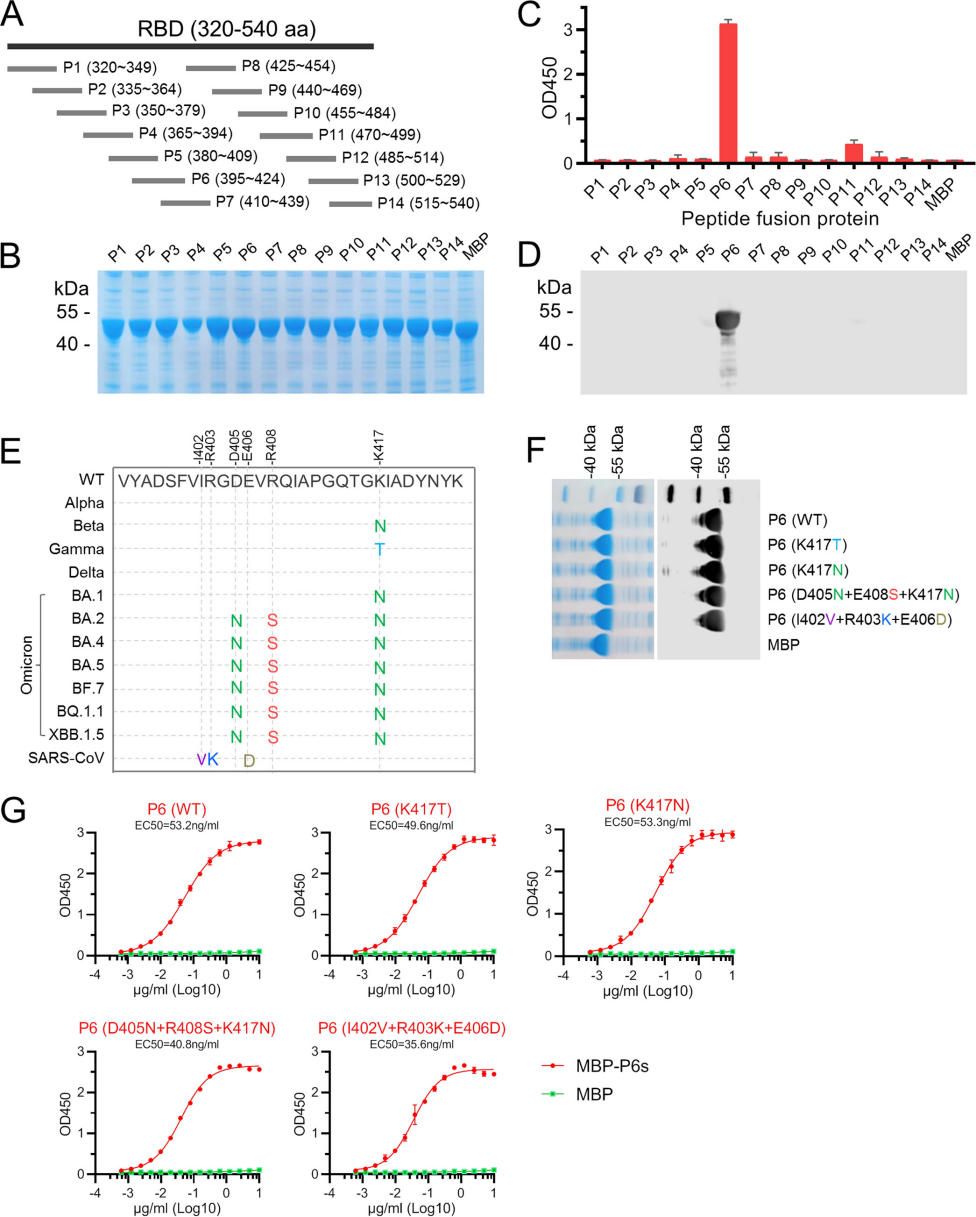
Epitope mapping and mutation. (A) Schematic diagram of the design of truncated overlapping short peptides spanning the RBD protein. (B) The short peptides were expressed as MBP-fusion proteins. The MBP-peptide fusion proteins were probed with MAb 22.9-1 by ELISA (C) and Western blotting (D). Peptide P6 was recognized by MAb 22.9-1. (E) The peptide P6 sequence was aligned with the corresponding sequence of the spike proteins of SARS-CoV-2 variants and SARS-CoV. (F and G) All five P6 mutants were expressed as MBP-fusion proteins (F) and probed with MAb 22.9-1 by Western blot analysis (F) and ELISA (G). Data from one of two independent experiments performed in triplicate are shown.

To map the antigenic epitopes of MAb 22.9-1, 14 partially overlapping short peptides, P1 to P14, were designed such that they spanned the entire length of the O-RBD protein (Fig. 4A). Of these, P1 to P13 were 30 amino acids in length, but P14 was 26 amino acids in length. The short peptides were expressed as maltose-binding protein (MBP) fusion proteins. Transformed Escherichia coli induced with isopropyl-β-d-thiogalactopyranoside (IPTG) expressed all 14 MBP-peptide fusion proteins in the soluble form (Fig. 4B). To identify the epitope of MAb 22.9-1, the MBP-peptide fusion proteins were probed with the MAb by ELISA. The results showed that P6 strongly reacted with MAb 22.9-1 (Fig. 4C). To verify the ELISA results, the MBP-peptide fusion proteins were further scanned by Western blot analysis, and the results were consistent with the results of ELISA: MAb 22.9-1 recognized P6 but not MBP or the other MBP-peptide fusion proteins (Fig. 4D). These results indicated that the epitope of MAb 22.9-1 located at P6 is ^395^VYADSFVIRGD(N)EVR(S)QIAPGQTGNIADYNYK^424^.

To determine whether MAb 22.9-1 can recognize more SARS-CoV-2 variants, spike protein sequences of SARS-CoV-2 VOCs and newly emerged strains, SARS-CoV, Middle East respiratory syndrome virus, human coronavirus, and animal coronavirus were aligned. The sequence homology comparison with peptide P6 is shown in Fig. 4E. Sequences with less than 50% homology with WT-P6 are not shown. Five P6 variant sequences were identified, and MBP fusion proteins of all five P6 mutants were obtained and probed with MAb 22.9-1. Western blot results showed that all five P6 mutants were recognized by 22.9-1 (Fig. 4F), and the binding ability of 22.9-1 for the P6 mutants was examined with ELISA. The results showed that MAb 22.9-1 broadly reacted with all P6 mutants with similar binding profiles and EC_50_s ranging from 35.6 ng/mL to 53.3 ng/mL (Fig. 4G). This result implied that the mutations at 405, 408, and 417 did not affect the binding of MAb 22.9-1 with the P6 mutants. These results also suggest that the P6 peptide was a conserved epitope in SARS-CoV-2 variants, and even in SARS-CoV.

### Fine-mapping of the epitope of 22.9-1.

**FIG 5:**
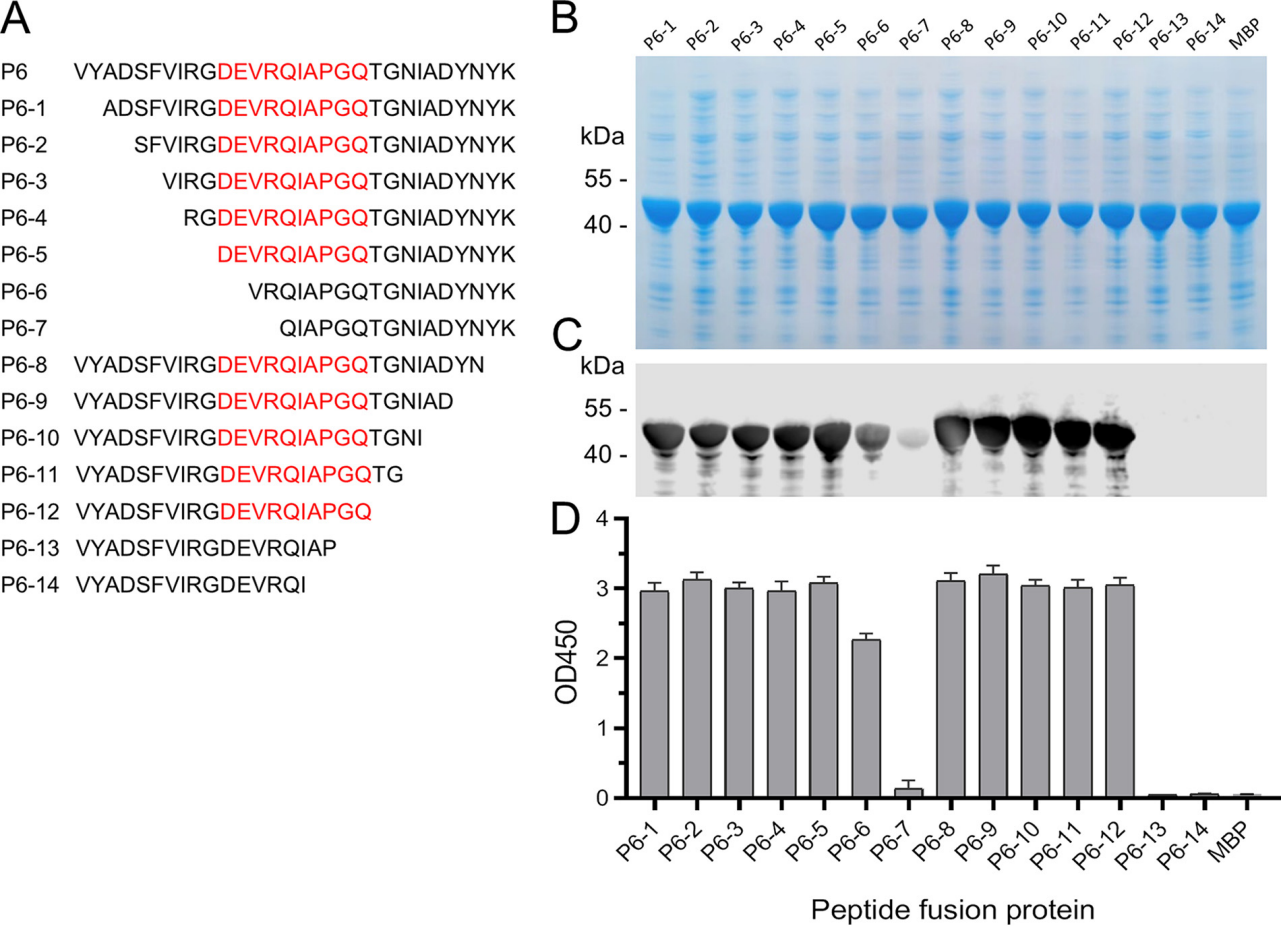
Identification of the core sequence of epitope 22.9-1. Peptide P6 was sequentially truncated from the amino or carboxy terminus as depicted in the schematic diagram (A). The truncated peptides were expressed as MBP-fusion proteins (B). Then, the peptides were probed with MAb 22.9-1 by ELISA (C) and Western blotting (D). The core sequence recognized by MAb 22.9-1 was determined to be ^405^DEVRQIAPGQ^414^.

To further refine the epitope of MAb 22.9-1, peptide P6 was sequentially truncated from the N terminus and the C terminus (Fig. 5A). The series of truncated peptides was expressed as MBP fusion proteins (Fig. 5B). The shortened peptide fusion proteins were probed with MAb 22.9-1 by ELISA and Western blotting. The results demonstrated that when no more than 10 amino acid residues were removed from the N terminus of P6, the reactivity between MAb 22.9-1 and the peptide fusion proteins was not significantly affected. However, when two more amino acid residues (^405^DE^406^) were removed, the reactivity between MAb 22.9-1 and P6-6 showed an obvious decrease (Fig. 5C and D). Next, when 10 or fewer amino acid residues were removed from the C terminus of P6, the peptide fusion proteins maintained strong reactivity with MAb 22.9-1, and when 12 amino acid residues were removed, the resulting fusion peptide nearly lost reactivity with MAb 22.9-1 (Fig. 5C and D). These results demonstrated that ^405^D(N)EVR(S)QIAPGQ^414^ represents the core conserved sequence of the linear epitope recognized by MAb 22.9-1.

### Location of the 22.9-1 epitope on the RBD.

**FIG 6:**
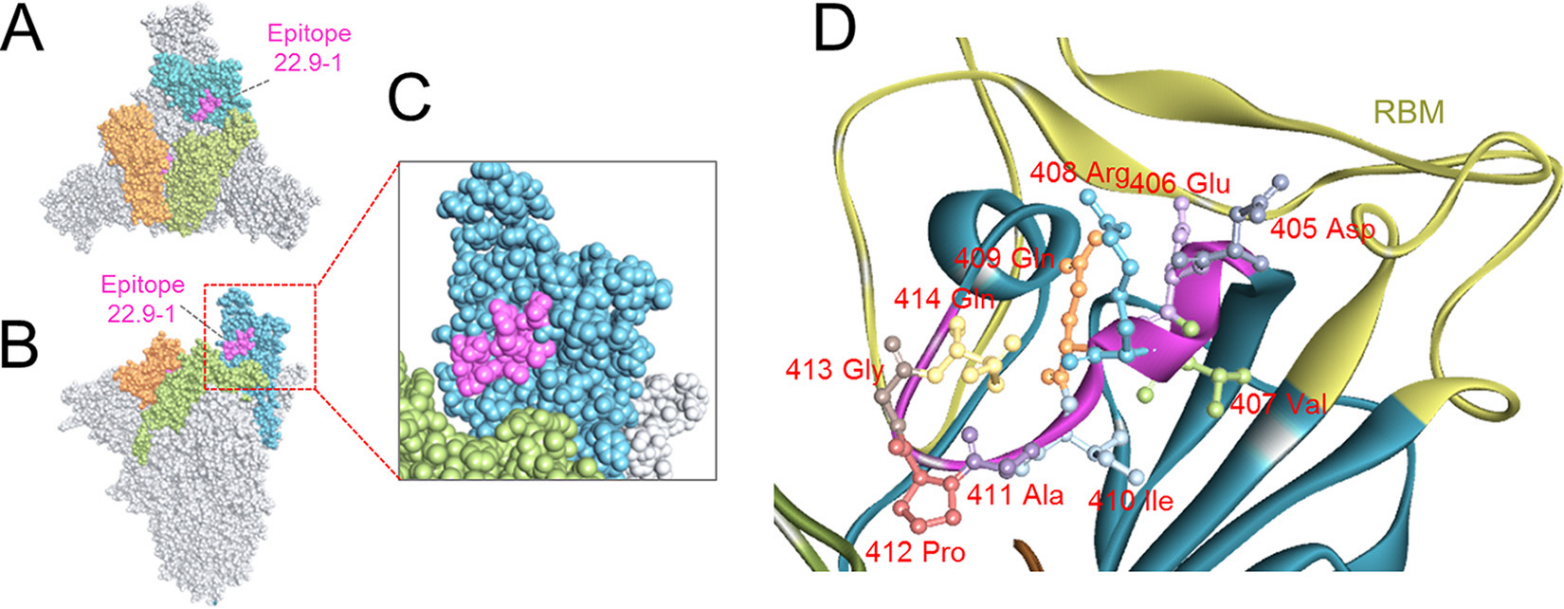
Visualization of epitope 22.9-1 in the S protein structure model. A stabilized SARS-CoV-2 S protein with one open-state RBD (PDB ID 7VRV) adopted to visualize the epitope. The NTD and S2 subunits are shown in gray, and the RBD domains are in cyan, light green, and orange. Epitope 405 to 414 is shown in purple. (A) Top view of the S protein trimer. (B) Side view of the S protein trimer. (C) Amplification of the epitope located in the RBD region on an open-state RBD. (D) Epitope 22.9-1 within the ribbon structure of the RBD. RBM is shown in copper, epitope 22.9-1 is shown in purple, and atoms of the amino residues are labeled with a unique ID and code.

The three-dimensional structure of the spike protein showed that epitope 22.9-1 is located on the internal surface of the RBD. The epitope was exposed on the up-state RBD, which facilitated the interaction of the antibody molecule with the epitope. In contrast, the epitope appeared to be buried, that is, it was not accessible, on the down-state RBD. Further, the epitope 22.9-1 formed a helical structure near the receptor-binding motif but did not overlap with it (Fig. 6).

## DISCUSSION

In the present study, a MAb (22.9-1) with good cross-binding abilities with RBD variants and broad neutralizing activities against SARS-CoV-2 variants was identified. In addition, a linear epitope of MAb 22.91 was identified within the internal surface of the RBD of the spike protein and found to be highly conserved in the Alpha, Beta, Gamma, and Delta variants of SARS-CoV-2. The antibody generated here and its epitope make an important contribution to the study on broad-spectrum neutralizing antibodies against and conserved epitopes of the SARS-CoV-2 virus.

We first generated seven neutralizing MAbs against the WT-S1 protein, of which five were specific to the RBD and recognized the same conformational epitope. However, none of the seven MAbs was able to neutralize the Omicron variant of the pseudovirus. These preliminary results confirmed that most neutralizing antibodies are susceptible to immune escape. Therefore, in the second round of monoclonal antibody preparation, we adopted a cross-selection ELISA strategy in which only antibodies that simultaneously reacted with WT-RBD, D-RBD, and O-RBD were selected. The results showed that only a very low percentage of clones were cross-reactive. Among hundreds of clones, only two clones were simultaneously cross-reactive with all three RBDs. These results suggested that the screening methods used to select broad-spectrum neutralizing antibodies are important.

We identified a highly conserved linear epitope of MAb 22.9-1 with the core sequence ^405^DEVRQIAPGQ^414^. Two mutation sites (D405N and R408S) in this core sequence have been identified in the recently emerged variants BA.2, BA.4, BQ.1.1, and XBB.1.5, as well as the BA.5.2 and BF.7 variants that have been dominant in China since November 2022 (18). Our peptide mutation analysis demonstrated that these two mutations did not influence the binding ability of MAb 22.9-1, as it showed strong neutralizing activities against the BA.2 variant (which harbors the D405N and R408S mutations in the S protein). Based on these findings, it can be concluded that MAb 22.9-1 has broad-spectrum neutralizing ability against nearly all the variants identified until now. Further, the neutralizing activities of MAbs against any variants that emerge in the future can be easily determined from the epitope sequence. Thus, the epitope sequence also provides useful information for further screening of broad-spectrum neutralizing antibodies and designing epitope-based vaccines. However, since MAb 22.9-1 is a murine monoclonal antibody, it needs to be humanized and further evaluated in future research.

The epitope ^405^D(N)EVR(S)QIAPGQ^414^ is located on the internal surface of the RBD and does not cover or overlap with the receptor binding motif (RBM). On the up-state RBD, the epitope is exposed for antibody binding, but on the down-state RBD, the epitope faces inwards and is hidden. However, we did not elucidate the structure of the antibody-antigen complex. Despite this, the epitope location results indicated that MAb 22.9-1 belongs to class 4 RBD-specific neutralizing antibodies, which recognize a cryptic epitope on the internal surface of the RBD and bind only to up-state RBDs (2). However, unlike class 1 to class 3 neutralizing antibodies, the neutralizing mechanism of class 4 neutralizing antibodies remains vague. Class 4 anti-RBD neutralizing antibodies mainly recognize a patch of conserved surface, and the neutralization may be mediated either by destabilization of the spike protein structure or by hindrance of ACE2 access (19–21). To evaluate whether MAb 22.9-1 can form a steric hindrance effect on the binding of RBD to ACE2, SPR experiments were performed through immobilizing ACE2 on a chip and flowover with RBD or RBD-MAb complexes. It was found that though MAb 22.9-1 did not completely block the binding of RBD to ACE2, it did interfere with the binding of RBD to ACE2 (Fig. S3). Although some cross-neutralizing antibodies targeting different conserved regions have been developed (22–26), more cross-neutralizing antibodies are needed to combat the emergence of SARS-CoV-2 VOCs. Combining neutralizing antibodies targeting different conserved regions could synergistically prevent the emergence of escape mutations (27). In this study, we provide a new conserved neutralizing target region that could be beneficial for epitope-based therapeutics and vaccine development.

### Conclusions.

In summary, we generated an RBD-specific MAb with broad neutralizing activity against SARS-CoV-2 variants, and we mapped a novel conserved linear neutralizing epitope on the RBD. This work provides important information for the development of broad-spectrum prophylactic and therapeutic strategies in the future.

## MATERIALS AND METHODS

### Cells, viruses, and sera.

The mouse myeloma cell line SP2/0 was maintained in RPMI (catalog number C11875500BT, Gibco), and BHK-21 cells, BSR-T7 cells, and Vero E6 cells were maintained in Dulbecco’s modified Eagle’s medium (DMEM; catalog number C11995500BT, Gibco) in a humidified 5% CO_2_ atmosphere at 37°C. All culture media were supplemented with 10% heat-inactivated fetal bovine serum (catalog number 10099-141, Gibco), 100 U/mL penicillin, and 100 μg/mL streptomycin (catalog number 15140122, Gibco).

### Protein expression and purification.

The SARS-CoV-2 S1 protein (WT) and three variants (WT, Delta, and Omicron) of the RBD constructs were generated as previously described (28, 29). Briefly, codon-optimized coding cDNA was synthesized and cloned into the SacI and XhoI sites of pCAGneo to generate the plasmids pCAG-opti-WT-S1, pCAG-opti-WT-RBD, pCAG-opti-D-RBD, and pCAG-opti-O-RBD. The 6× His tag sequence was added at the carboxyl terminal of each protein. The resulting plasmids were linearized with SspI and used to transfect BHK-21 cells to generate stable expression cell lines. Stable cell lines were constructed and selected as previously described (28, 29). Briefly, BHK-21 cells were transfected with linearized plasmids using the FuGENE HD transfection reagent (catalog number 4709705001, Roche). After transfection, cells were cloned and selected with G418. The cell clones were identified by indirect IFA and Western blot assay with 6× His tag-specific mouse monoclonal antibody. The His-tagged proteins were purified from supernatants of the constructed stable cell lines by affinity chromatography with Ni-nitrilotriacetic acid resin.

### Generation of MAbs.

Purified recombinant S1 or RBD protein was used as an immunogen in mice, and hybridomas secreting anti-S1 or anti-RBD antibodies were generated according to the procedure described previously (30). Briefly, 6-week-old, female BALB/c mice were immunized subcutaneously with recombinant protein emulsified with Freund’s complete adjuvant (Sigma). The mice were then given two booster injections of recombinant protein emulsified with Freund’s incomplete adjuvant. A final intraperitoneal booster immunization of protein without adjuvant was given 3 days prior to the harvest of spleen cells for hybridoma fusion. The fused cells were cultured and selected in hypoxanthine-aminopterin-thymidine (HAT) medium. Cell culture supernatants from the surviving clones were screened by indirect ELISA, and positive cell lines were subcloned three times with the limiting dilution method. Ascitic fluid was generated in pristane-primed BALB/c mice. The monoclonal antibodies were purified by affinity chromatography in a HiTrap Protein G HP column (catalog number 29048581, GE) according to the manufacturer’s instructions. The heavy and light chains of each MAb were isotyped with a Pierce rapid isotyping kit with kappa and lambda mouse Ig (catalog number 26179, Thermo).

### IFA.

S1- or RBD-expressing cell lines or BHK-21 cells were fixed with 4% paraformaldehyde for 20 min at room temperature, permeabilized with 0.1% Triton X-100 in phosphate-buffered saline (PBS) at 4°C for 10 min, and incubated with 4% bovine serum albumin in PBS at 37°C for 30 min. Cells were first incubated with monoclonal antibodies at 37°C for 1 h, and this was followed by incubation with fluorescein-conjugated goat anti-mouse IgG (catalog number ZF-0312, ZSGB-BIO) at room temperature for 1 h. In the final step, nuclei were stained with 4′,6-diamidino-2-phenylindole for 15 min at room temperature. The cells were then washed with PBS three times and visualized by fluorescence microscopy.

### ELISA.

For detection of the binding ability of the obtained monoclonal antibodies with SARS-CoV-2 S1 or RBD variants, 96-well polystyrene microplates were coated with purified S1 or RBD variants at a concentration of 2 μg/mL in 0.05 M carbonate buffer (pH 9.6) at 4°C overnight and blocked with 5% skimmed milk for 2 h at 37°C. The microplates were then washed three times with PBS plus 0.5% Tween 20 (PBST), and 100 μL of hybridoma cell supernatant or diluted MAbs sample was added and incubated for 1 h at 37°C. The plates were, again, washed three times with PBST and incubated with horseradish peroxidase (HRP)-conjugated goat anti-mouse IgG (catalog number ZB-2305, ZSGB-BIO) for 1 h at 37°C. This was followed by three more washes with PBST, after which 100 μL of 3,3′,5,5′-tetramethyl benzidine (TMB) was added to the plates. The reaction was terminated with 2 M H_2_SO_4_, and optical density was measured at 450 nm (OD_450_) using an ELISA plate reader. The data were analyzed using the GraphPad Prism software.

For blocking ELISA, MAb 20.8-1 was used as the detecting antibody and was conjugated with HRP using the EZ-Link Plus activated peroxidase kit (Thermo Scientific) according to the manufacturer’s instructions. Microplates were coated with 2 μg/mL S1 protein and blocked with skimmed milk. The plates were then washed with PBST, and 100 μL of hybridoma cell supernatants was added and incubated for 1 h at 37°C. The plates were washed with PBST again and incubated with 100 μL HRP-conjugated detecting antibody for 30 min at 37°C. After another wash with PBST, the TMB substrate was added. The reaction was terminated with 2 M H_2_SO_4_, and the OD_450_ was detected.

For determining the reactivity of peptides with MAbs, 96-well polystyrene microplates were coated with peptide-MBP fusion proteins and then blocked with skimmed milk. The plates were washed with PBST, and diluted MAb samples were added and incubated for 1 h at 37°C. The plates were washed with PBST, incubated with HRP-conjugated goat anti-mouse IgG, washed with PBST again, and incubated with the TMB substrate. The reaction was terminated with 2 M H_2_SO_4_, and the OD_450_ was detected. Vector-expressed MBP protein was used as a negative control. The results depict the average value from experiments that were conducted in triplicate.

### Neutralization assay.

The VSVΔG-GFP plasmid was pseudotyped with recombinant SARS-CoV-2 spike protein to generate pVSVΔG-GFP-nCoVS, as previously described (31, 32). Briefly, SARS-CoV-2 S genes were chemically synthesized and cloned into the plasmid pVSVΔG-GFP to produce pVSVΔG-GFP-nCoVS. Four variants of the S gene were used to generate the pseudovirus: WT (GenBank accession number NC_045512), Delta (GISAID, EPI_ISL_2863933), Omicron BA.1 (BA.1, GISAID, EPI_ISL_7160037), and Omicron BA.2 (BA.2, GISAID, EPI_ISL_15517344). pVSVΔG-GFP-nCoVS containing the VSV N, P, and L protein-expressing plasmids was transfected into BSR-T7 cells. At posttransfection 96 h, the supernatants were harvested. The rescued viruses were passaged and titers were determined on Vero E6 cells.

For the neutralization assay, monoclonal antibodies were 2-fold serially diluted with DMEM in 96-well microplates. Then, 100 50% tissue culture infective doses of VSVΔG-GFP-nCoVS in 50 μL was added into each well and incubated at 37°C for 1 h. After the neutralization assay, Vero E6 cells were added into microplates at a density of 3 × 10^5^ cells per well, incubated (to facilitate infection) for 24 h at 37°C in a 5% CO_2_ atmosphere, and then stained with Hoechst 33342. The cells were imaged using a fluorescence microscope, and the rate of infection was quantified by automated enumeration of the total number of cells and those expressing green fluorescent protein (GFP) by using the Image J software. The rate of infection was normalized to the average number of cells infected with VSVΔG-GFP-nCoVS incubated with diluent control. Data are presented as the relative infection inhibition rate for each antibody concentration. Neutralization EC_50_ titers were calculated using GraphPad Prism.

### Measurement of antibody-binding affinity by SPR.

The binding affinity of the MAb 22.9-1 for the RBDs of the WT, Delta, and Omicron variants were determined by SPR binding assays with the Biacore 8K instrument and protein A chip (Cytiva). HBS-EP^+^ buffer (Cytiva) was used as the running buffer and sample diluent. Monoclonal antibody was captured over the protein A surface for 60 s at a flow rate of 10 μL/min. The RBD antigens were injected into each cycle at a flow rate of 30 μL/min; this was followed by contact for 120 s and dissociation for 400 s. The chip surface was regenerated each cycle with glycine solution (pH 1.5) at a flow rate of 30 μL/min for 30 s. The data were analyzed, and affinity *K_D_* values were calculated with the Biacore Insight Evaluation software.

### SPR-based ACE2 competition experiments.

To determine the neutralization mechanism of MAb 22.9-1, binding competition experiments were conducted by SPR assay with the Biacore 8K instrument and NTA chip (Cytiva). Purified hACE2 (Sino Biological) was immobilized at a concentration of 10 μg/mL. The following samples were injected: (i) 40 μg/mL O-RBD, (ii) complex of 40 μg/mL O-RBD and 0.15 mg/mL MAb 22.9-1, (iii) complex of 40 μg/mL O-RBD and 0.15 mg/mL MAb 20.8-8. The data were analyzed with the Biacore Insight Evaluation software. The curves were plotted using GraphPad Prism software.

### Expression of short peptide-fusion proteins.

To map the epitope of the selected MAb 22.9-1, a set of overlapping short peptides spanning the whole WT-RBD was designed. Short peptides were expressed as fusion proteins with MBP in the pMAL-c5X plasmid. Peptides encoding DNA fragments were synthesized and cloned into the NdeI and BamHI sites of pMAL-c5X. The resulting recombinant plasmids were verified by DNA sequencing. The verified recombinant plasmids were transformed into Escherichia coli ER2523, and expression of the proteins was induced with IPTG at a final concentration of 0.3 mM for 3 h at 30°C. The expressed proteins were analyzed with SDS-PAGE. Some recombinant proteins were purified by MBPTrap HP column affinity chromatography according to the manufacturer’s instructions.

### SDS-PAGE and Western blotting.

Cell lines expressing the recombinant proteins or bacteria expressing the peptide-infused proteins were subjected to 4% to 20% SDS-PAGE (GenScript catalog number M42015C) for protein separation, transferred onto a nitrocellulose membranes, and blocked with 5% skimmed milk at 4°C overnight. The membranes were then probed with MAb 22.9-1 at 37°C for 1 h, washed three times with PBST, incubated with anti-mouse Alexa Fluor 680-conjugated secondary antibodies (catalog number A10038, Thermo) or HRP-conjugated goat anti-mouse IgG for 1 h at 37°C, and then washed three times with PBST. Protein bands were detected with the Li-Cor Odyssey system (Li-Cor Biosciences, Lincoln, NE, USA) or developed with the enhanced chemiluminescence substrate.

### Structure analysis of the neutralizing antibody-recognizing epitope.

Discovery Studio (BIOVIA) software was used to visualize the structures of SARS-CoV-2 S proteins (PDB 7VRV) corresponding to various up/down states of three RBD protomers. Details of the antibody recognition sites are displayed from different perspectives in Fig. 6.

### Ethics statement.

All animal experiments of this study were approved by the Ethics and Animal Welfare Committee of Harbin Veterinary Research Institute. All mice used in this study were carefully fed, and suffering of animals was minimized.

### Data availability.

We confirm that the data supporting the findings of this study are available within the article and its supplemental material.
