# Supplementary material for: A Novel Conserved Linear Neutralizing Epitope on the Receptor-Binding Domain of the SARS-CoV-2 Spike Protein
Source: Microbiol Spectr. 2023 Jun 12;11(4):e01190-23. doi: 10.1128/spectrum.01190-23 (PMC10433833; doi:10.1128/spectrum.01190-23)
Supplement: Supplemental file 1 — Fig. S1 to S3. Download spectrum.01190-23-s0001.docx, DOCX file, 0.8 MB [file spectrum.01190-23-s0001.docx]

**Supplementary Data**

**A novel conserved linear neutralizing epitope on the receptor-binding domain of the SARS-CoV-2 spike protein**

Rong-Hong Hua^1,*^, Shu-Jian Zhang^1^, Bei Niu^1^, Jin-Ying Ge^1^, Ting Lan^1^, Zhi-Gao Bu^1,*^

^1^ State Key Laboratory for Animal Disease Contgrol and Prevention, Harbin Veterinary Research Institute of Chinese Academy of Agricultural Sciences, Harbin, 150069, China.

*Corresponding author:

Rong-Hong Hua, [huaronghong@caas.cn](mailto:huaronghong@caas.cn), ORCID: 0000-0001-7034-5766

Zhi-Gao Bu, [buzhigao@caas.cn](mailto:buzhigao@caas.cn), ORCID: 0000-0001-9242-4211

**
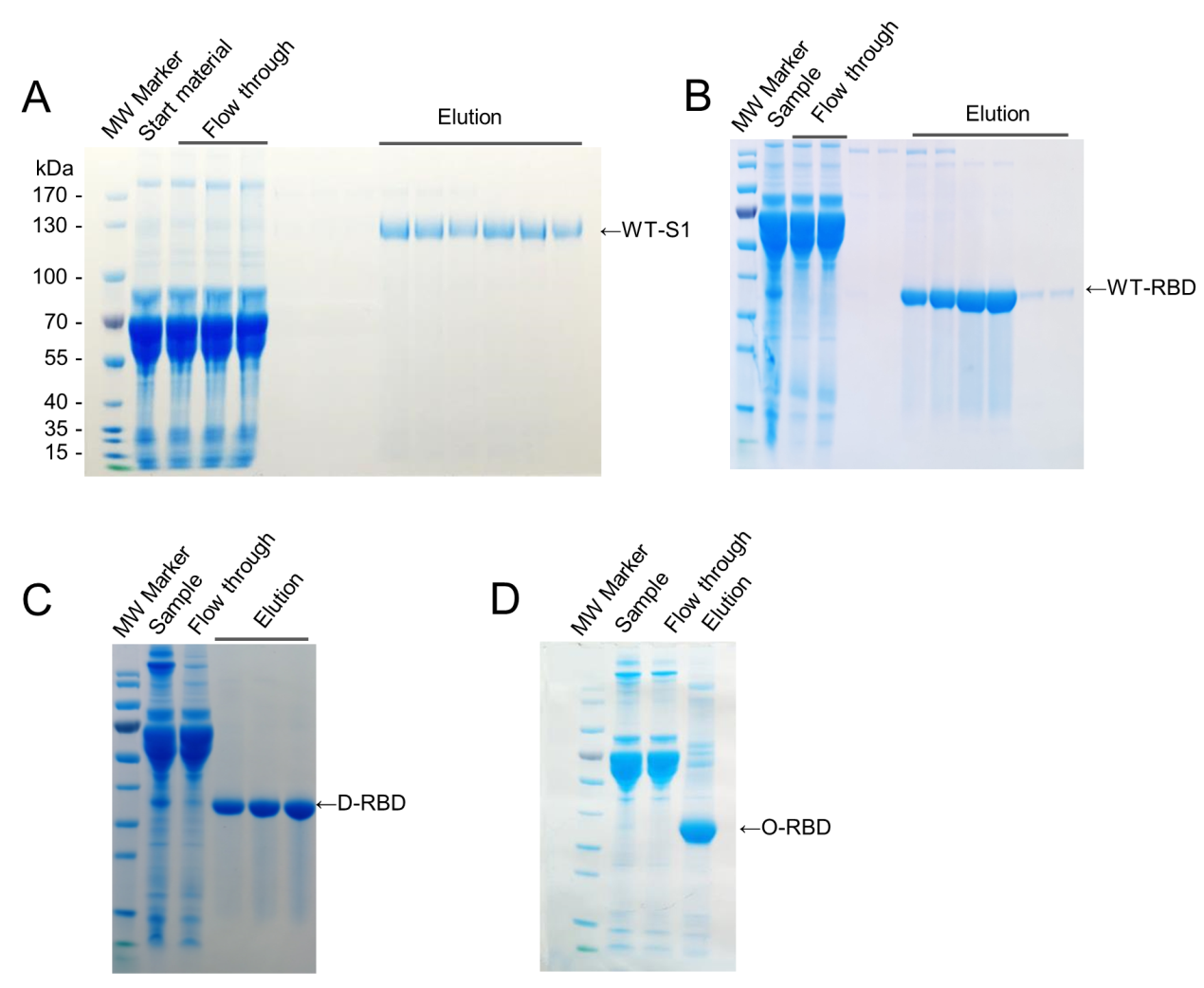
**

**Fig S1. Protein expression and purification.** After transfection and selection, stable cell lines were generated. The supernatants of cell lines were used to purify the expressed protein by Ni-affinity chrommtography. The purified proteins were subjected to SDS-PAGE analysis. The results showed that WT-S1 (A), WT-RBD (B), D-RBD (C) and O-RBD (D) were all well purified.


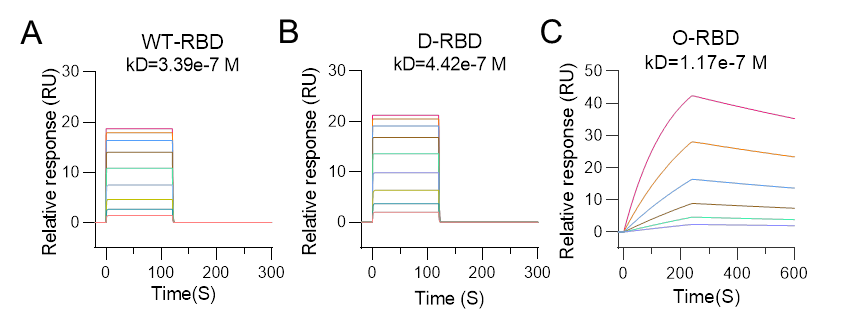


**Fig S2. Measurement of antibody-binding affinity by SPR.** The binding affinity of the mAb 22.9-1 for the WT-RBD (A), Delta-RBD (D-RBD) (B), and Omicron-RBD (O-RBD) (C) were determined by SRP binding assays with the Biacore 8K instrument and protein A chip.

**Fig S3. mAb and ACE2 competition experiment by SPR.** ACE2 was immobilized on a chip, and then O-RBD or complexes of O-RBD with either mAb 22.9-1 or mAb 20.8-8 flowed over.
